# Dosimetry Results from the Phase 1b/3 ACTION-1 Trial of [^225^Ac]Ac-DOTATATE (RYZ101) in Patients with Somatostatin Receptor–Expressing, Well-Differentiated GEP-NETs

**DOI:** 10.2967/jnumed.125.270573

**Published:** 2026-08

**Authors:** George Sgouros, Gary A. Ulaner, Bin He, Michael Ghaly, Eric Frey, Sandy Kotiah, Aaron Scott, Denis Ferreira, Eileen Sneeden, Michael Morris

**Affiliations:** 1 Russell H. Morgan Department of Radiology and Radiological Science, School of Medicine, Johns Hopkins University, Baltimore, Maryland;; 2 Radiopharmaceutical Imaging and Dosimetry, LLC (Rapid), Baltimore, Maryland;; 3 Molecular Imaging and Therapy, Hoag Family Cancer Institute, Irvine, California;; 4 Departments of Radiology and Translational Genomics, University of Southern California, Los Angeles, California;; 5 Mercy Medical Center, Baltimore, Maryland;; 6 United Theranostics, Glen Burnie, Maryland;; 7 RayzeBio, San Diego, California; and; 8 Institute of Nuclear Medicine, Bethesda, Maryland

**Keywords:** ^225^Ac, targeted α-therapy, DOTATATE, dosimetry, GEP-NETs, RYZ101

## Abstract

Gastroenteropancreatic neuroendocrine tumors (GEP-NETs) are biologically and clinically heterogeneous tumors, most of which overexpress somatostatin receptors (SSTRs). The presence of SSTRs enables staging and allows patient selection for treatment with radiolabeled somatostatin analogs (SSAs), such as DOTATATE. [^225^Ac]Ac-DOTATATE is an α-emitting radiopharmaceutical in development for the treatment of patients with SSTR2-expressing solid tumors. α particles have a shorter path length and higher linear energy transfer than do β particles, leading to largely irreparable double-strand DNA breaks and cytotoxicity. ACTION-1 (NCT05477576) is a 2-part, randomized, controlled, open-label, phase 1b/3 trial of [^225^Ac]Ac-DOTATATE in patients with SSTR-expressing GEP-NETs whose disease progressed after ^177^Lu-labeled SSA therapy. ACTION-1 included a dosimetry substudy to determine the feasibility of obtaining imaging data with ^225^Ac. The objectives of the substudy were to estimate radiation absorbed doses of [^225^Ac]Ac-DOTATATE in critical organs and tumors. **Methods:** [^225^Ac]Ac-DOTATATE was administered intravenously every 8 wk for 1–4 cycles to adults with grade 1 or 2, well-differentiated, inoperable SSTR2-expressing GEP-NETs that progressed after 2–4 cycles of ^177^Lu-lableled SSA. Dosimetry was assessed after cycles 1 and 4 of [^225^Ac]Ac-DOTATATE via SPECT/CT of ^221^Fr (4.8-min half-life), the first daughter and surrogate for ^225^Ac and ^213^Bi (46.6-min half-life, surrogate for later daughters). Absorbed doses and absorbed dose coefficients adjusted for a relative biological effectiveness factor of 5 (ADC_RBE=5_) to target tissues and tumors were calculated; tumor doses were estimated by MIRD *S*-value methodology. **Results:** Dosimetry data were obtained from 9 of 17 patients in phase 1b, each completing 4 cycles. ^213^Bi mostly remained with DOTATATE in the tumors; a minor fraction went to the kidneys. The estimated absorbed doses to kidneys and red bone marrow were 22.3 and 1.1 Gy, respectively. The ADC_RBE=5_ to selected tumors ranged from 488 to 8775 mGy/MBq across both treatment cycles. The ADC_RBE=5_ for most tumors was lower in cycle 4 than in cycle 1; many tumors defined in cycle 1 were unidentifiable in cycle 4. **Conclusion:** Dosimetry data from ACTION-1 demonstrated the feasibility of image-based dosimetry of ^225^Ac through SPECT/CT imaging of ^221^Fr and ^213^Bi. The ^213^Bi daughter mostly remained with DOTATATE in tumors, decaying at the same location as ^221^Fr. The favorable tumor–to–normal tissue absorbed dose ratio of [^225^Ac]Ac-DOTATATE supports its use in patients with SSTR-expressing GEP-NETs.

## Visual Abstract

**Figure:**
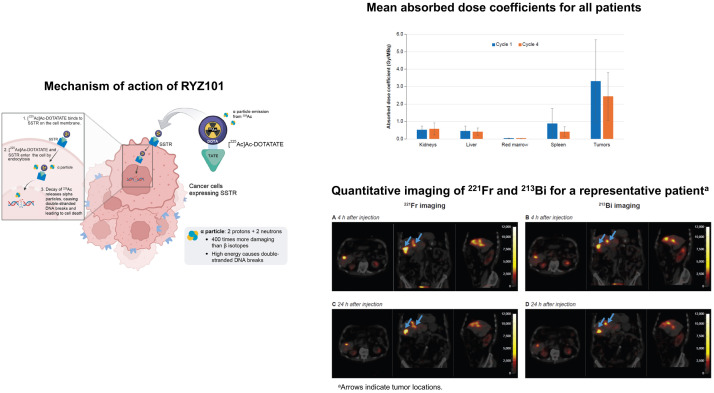

Gastroenteropancreatic neuroendocrine tumors (GEP-NETs) are biologically and clinically heterogeneous neoplasms arising from neuroendocrine precursor cells in the gastrointestinal tract and pancreas (1,2). An estimated 40% of patients with GEP-NETs present with regionally advanced or metastatic disease (3) requiring nonsurgical interventions. Well-differentiated GEP-NETs are commonly characterized by high-density expression of somatostatin receptors (SSTRs), which can be targeted by radiopharmaceutical therapy with radiolabeled somatostatin analogs (SSAs) (4–6).

Peptide receptor radiopharmaceutical therapy with [^177^Lu]Lu-DOTATATE is recommended for patients whose tumors have progressed on SSAs (7,8). In NETTER-1, the median progression-free survival time was statistically significantly better in patients receiving [^177^Lu]Lu-DOTATATE versus the control group (hazard ratio, 0.18; 95% CI, 0.11–0.29; *P* < 0.0001) (8).

Despite the prolonged progression-free survival time reported in NETTER-1, patients eventually progress after ^177^Lu therapy and need further treatment. Treatment with ^225^Ac could overcome resistance to [^177^Lu]Lu-DOTATATE, as ^225^Ac is an α-emitting isotope with high linear energy transfer that causes DNA double-strand breaks (9). [^225^Ac]Ac-DOTATATE is a first-in-class, highly potent α-emitting radiopharmaceutical being developed for the treatment of patients with SSTR2-expressing solid tumors. After the binding of [^225^Ac]Ac-DOTATATE to SSTRs, α-particle emissions from ^225^Ac lead to irreparable DNA double-strand damage and cytotoxicity (10). In 1 clinical study, treatment with [^225^Ac]Ac-DOTATATE resulted in partial responses in 15 of 32 patients with GEP-NETs and stable disease in 9 patients (11). In a longer-term real-world study of 91 patients receiving 453 cycles of [^225^Ac]Ac-DOTATATE, the median overall survival was not reached after a median follow-up of 24 mo, and 44% of patients had complete or partial morphologic responses (12).

The phase 1b/3 ACTION-1 trial compared [^225^Ac]Ac-DOTATATE with the standard of care in patients with SSTR-expressing, well-differentiated GEP-NETs that progressed after prior ^177^Lu-labeled SSA therapy. Here, we report the results of a dosimetry substudy conducted during phase 1b of ACTION-1 to determine the feasibility of obtaining imaging data with ^225^Ac.

## MATERIALS AND METHODS

### Study Design and Patients

**FIGURE 1.**
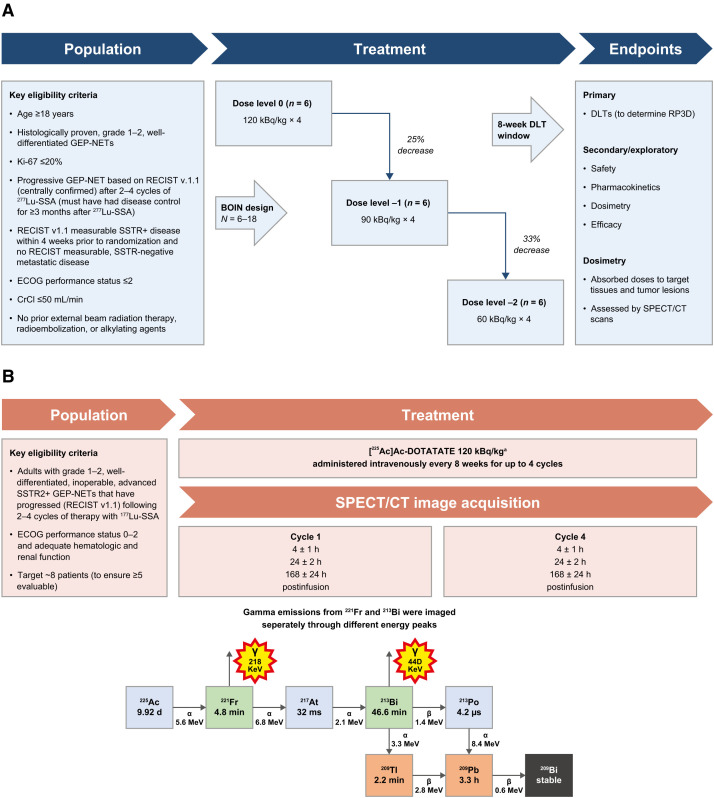
ACTION-1 study design. (A) Phase 1b methods. (B) Dosimetry substudy methods. Supplemental Table 1 provides SPECT/CT timepoints used for patients. BOIN = Bayesian optimal interval design; CrCl = creatinine clearance; DLT = dose-limiting toxicity; ECOG = Eastern Cooperative Oncology Group; RP3D = recommended phase 3 dose.

ACTION-1 is a global, randomized, controlled, open-label phase 1b/3 trial of [^225^Ac]Ac-DOTATATE in patients with SSTR-expressing, well-differentiated GEP-NETs (NCT05477576). Phase 1b (Fig. 1) was a dose de-escalation study using Bayesian optimal interval design (13,14), with the primary objective of determining the recommended phase 3 [^225^Ac]Ac-DOTATATE dose. The secondary objectives were to investigate the safety, pharmacokinetics, dosimetry, and efficacy of [^225^Ac]Ac-DOTATATE.

Adults with grade 1 or 2, well-differentiated, inoperable, advanced SSTR2-expressing GEP-NETs that progressed (RECIST version 1.1) after 2–4 cycles of ^177^Lu-labeled SSA were eligible. Detailed eligibility criteria and study design details can be found in the supplemental materials, available at http://jnm.snmjournals.org (9,15–27).

The study design was approved by institutional review boards or independent ethics committees at each study site and complied with International Ethical Guidelines for Biomedical Research involving Human Subjects, Good Clinical Practice guidelines, and the Declaration of Helsinki. All patients provided written informed consent.

### Treatment and Image Analysis

Treatment and image analysis are detailed in the supplemental materials. In brief, [^225^Ac]Ac-DOTATATE was administered intravenously every 8 wk for 1–4 cycles. Lysine and arginine were coinfused with each [^225^Ac]Ac-DOTATATE administration for kidney protection.

SPECT/CT acquisitions were performed with a high-energy collimator and appropriate uniformity maps. Energy windows were centered over ^225^Ac photopeaks at 218.2 and 440 keV. The single field of view was set from the liver, including the kidneys and the lumbar spine to L5, as well as any tumors in the field of view.

Reconstructed images were converted to activity concentration units using calibration phantom scan results. A calibration factor was obtained for each imaging system used for this trial during site qualification. Calibration was performed by imaging a phantom with known ^225^Ac activity in a sphere; there was no activity in the phantom background. The activity placed in the sphere was produced several days before it was placed in the phantom. ^221^Fr and ^213^Bi were thus in secular equilibrium with ^225^Ac activity. SPECT/CT data were reconstructed to give ^221^Fr and ^213^Bi reconstructions. A volume of interest was defined over the sphere in the reconstructed image, which was larger than the sphere to compensate for partial-volume effects. The sum of voxel values in each volume of interest was used to calculate respective radionuclide-specific activity concentrations. The same volume of interest was used for activity and mass. No special partial-volume compensation was performed. Images used for activity quantification were reconstructed, with compensation for full collimator–detector responses and 40 iterations of 24 subsets.

Organ doses were estimated using SPECT/CT scans obtained at 4, 24, and 168 h after [^225^Ac]Ac-DOTATATE injection (Supplemental Table 1). Activity was quantified in the liver, both kidneys, spleen, red marrow (L3−L5 vertebrae), and up to 5 tumors. The tumor selection criterion was [^225^Ac]Ac-DOTATATE uptake greater than the surrounding/adjacent background tissue at all available timepoints, preferably with the longest diameter exceeding 2 cm.

Organ and lesion time–activity curves for ^221^Fr and ^213^Bi were fitted to either monoexponential decay or exponential uptake, with washout functions dependent on the shape of the time–activity curve. The time-integrated activity coefficient was calculated as time-integrated activity divided by administered activity. All time-integrated activity coefficients were calculated using 3D-RD-S (Rapid, LLC). Inputs to 3D-RD-S were the time–activity curves for each source tissue; output time-integrated activity coefficients were used to calculate absorbed doses.

Absorbed doses and absorbed dose coefficients (ADCs) to target tissues and tumors were calculated using MIRD Committee *S*-value methodology (15,16,25), with mass corrections implemented inside 3D-RD-S. Particle yields and energies used for absorbed dose calculations were obtained from International Commission on Radiological Protection publication 107 (18). A relative biological effectiveness (RBE) factor of 5 was applied to the α dose contributions (9). Doses from ^225^Ac and its daughters were separately calculated and summed in accordance with abundances with the following assumptions: activity distributions for ^225^Ac and ^217^At (half-life, 32 ms) were assumed to be the same as that of ^221^Fr (half-life, 4 min), given their short half-lives. The distribution of ^213^Bi daughters was assumed to be the same as that of ^213^Bi, given the short half-lives of ^213^Po (4.2 µs; accounts for 28.9% of the energy emitted) and ^209^Tl (2.2 min; 0.2% energy emitted), with ^209^Pb accounting for only 0.7% of emitted energy. More details on the energy emitted and decay half-lives are presented in Supplemental Table 2 (18).

Tumor doses were estimated using MIRD *S*-value methodology, including only self-dose, using 3D-RD-S. Calculations used *S* values for spheres of various sizes on the basis of specific absorbed fractions data (23). The sphere having the closest volume to each tumor was used, and mass correction was applied to account for residual size difference.

### Statistical Analysis

All analyses were descriptive. Continuous variables were summarized as means and SDs or medians with ranges.

## RESULTS

### Patients

In the phase 1b of ACTION-1, 17 patients received at least 1 dose of [^225^Ac]Ac-DOTATATE at the starting dose of 120 kBq/kg. The dosimetry study cohort comprised 9 patients at 2 sites in the United States (7 men and 2 women; median age, 58 y [range, 42–76 y]) who completed cycles 1 and 4 (Table 1).

**TABLE 1.** Patient Characteristics at Baseline (*n* = 9)

Characteristic	Value
Age (y)	58 (42–76)
Sex	
Male	7 (77.8)
Female	2 (22.2)
Race	
White	7 (77.8)
Black or African American	1 (11.1)
Unknown	1 (11.1)
Ethnicity	
Hispanic or Latino	1 (11.1)
Not Hispanic or Latino	8 (88.9)
ECOG performance status	
0	8 (88.9)
1	1 (11.1)
Duration of GEP-NET (y)	7.9 (1.3–19.1)
Primary tumor site	
Ileum	4 (44.4)
Pancreas	4 (44.4)
Duodenum	1 (11.1)
Functional status	
Functional	5 (55.6)
Not functional	4 (44.4)
Histopathology grade	
1	5 (55.6)
2	4 (44.4)

ECOG = Eastern Cooperative Oncology Group.

All patients received 4 cycles of [^177^Lu]Lu-DOTATATE cycles before this study, with a median time from treatment to the first dose of [^225^Ac]Ac-DOTATATE of 27.1 mo (range, 1.9–45.8 mo).

Qualitative data expressed as number, followed by percentage in parentheses; continuous data expressed as median, followed by range in parentheses.

### Dosimetry

The mean ADC adjusted for an RBE factor of 5 (ADC_RBE=5_) values for the kidneys, liver, red marrow, and spleen were 519.8 ± 224.7, 453.6 ± 279.5, 28.6 ± 12.1, and 882.2 ± 424.4 mGy/MBq, respectively, in cycle 1 and 575.2 ± 352.6, 415.4 ± 220.7, 24.6 ± 14.1, and 862.5 ± 281.4 mGy/MBq, respectively, in cycle 4. Summary statistics for ADC_RBE=5_ to target tissues for 9 patients in cycles 1 and 4 are provided in Supplemental Table 3.

The ADC_RBE=5_ to 40 selected tumors (25 in cycle 1 and 15 in cycle 4) ranged from 488 to 8775 mGy/MBq across both treatment cycles (Supplemental Table 4). Most tumor absorbed doses were lower in cycle 4 than in cycle 1; 10 tumors identified in cycle 1 were not identifiable in cycle 4.

**FIGURE 2.**
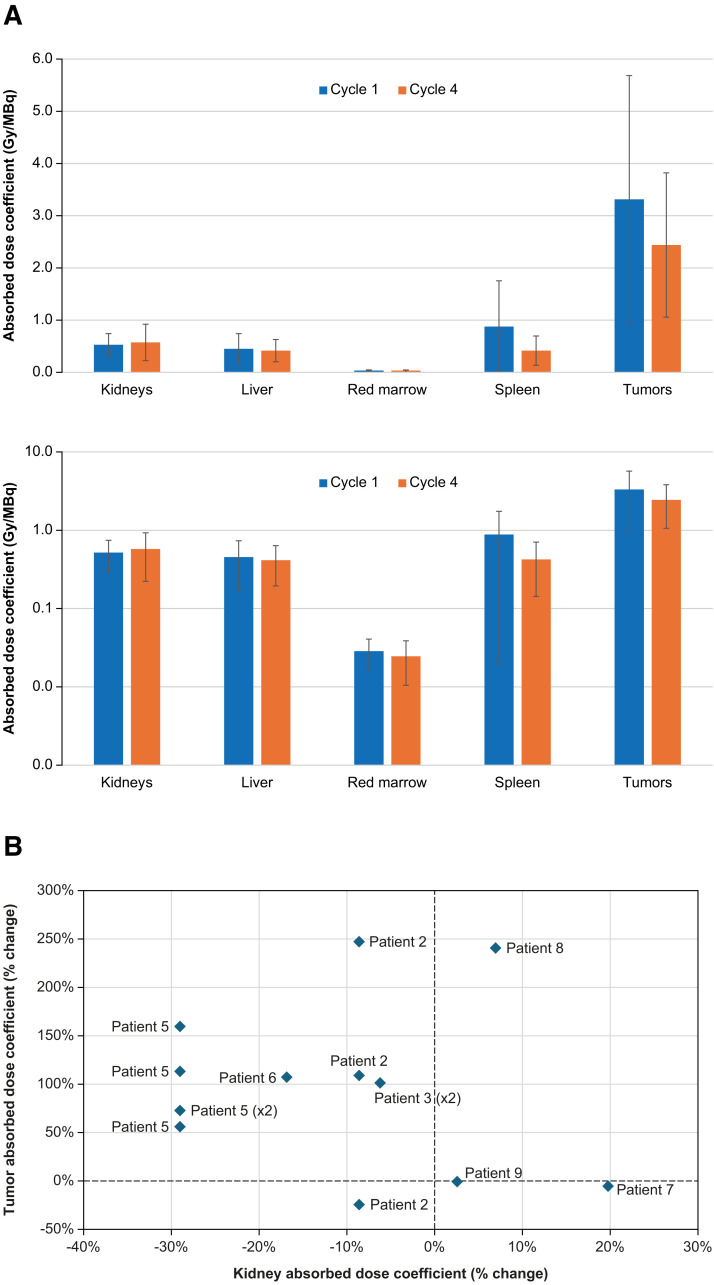
(A) Mean ADC_RBE=5_ by cycle for all patients (*n* = 9) displayed on linear (top panel) and logarithmic (bottom panel) scales. Error bars represent SD. (B) Changes in ADC_RBE=5_ between cycles 1 and 4, calculated as (cycle 1 kidney or tumor lesion absorbed dose coefficient − cycle 4 kidney or tumor lesion ADC_RBE=5_)/(cycle 4 kidney or tumor ADC) × 100%. Data are shown only for patients whose tumors were defined in both cycles 1 and 4.

The mean ADC_RBE=5_ decreased from cycle 1 to cycle 4, except in the kidneys, where a small increase was observed (cycle 1, 519.8 ± 224.7 mGy/MBq; cycle 4, 575.2 ± 352.6 mGy/MBq) (Fig. 2A). A reduction in kidney ADC_RBE=5_ was generally accompanied by an increase in tumor ADC (Fig. 2B). No apparent trends for absorbed doses to normal organ tissue were observed.

In both cycles, 14 tumors were identified in 7 of 9 patients, 3 of which (patients 2, 7, and 9) had a lower ADC_RBE=5_ in cycle 1 than in cycle 4 (Fig. 2B). Three participants (patients 7, 8, and 9) had a higher kidney ADC_RBE=5_ in cycle 1, each of whom had 1 or 2 identified tumors (Supplemental Table 4), indicating lower tumor burden. In patient 2, 1 of 3 tumors had a higher ADC_RBE=5_ in cycle 4 and 2 had a lower ADC_RBE=5_. Only patient 8 had a lower ADC_RBE=5_ in cycle 4 for both the kidney and tumor.

**FIGURE 3.**
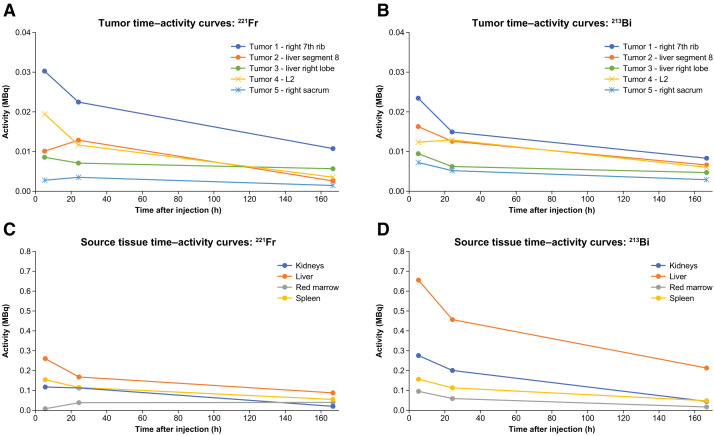
Tumor and normal tissue time–activity curves ^221^Fr and ^213^Bi for representative patient (patient 1) over cycle 1. Supplemental Figures 1 and 2 show plots of time–activity curves for ^225^Ac and ^213^Bi in individual organs.

Estimated time–activity curves for ^221^Fr and ^213^Bi are shown in Figure 3 for a representative patient (patient 1). The activities of ^221^Fr and ^213^Bi were generally comparable in the tumor, although ^213^Bi activity was numerically greater than that of ^221^Fr in the kidneys and liver. Summary statistics for time-integrated activity curves (Table 2) indicated that ^213^Bi was retained at the tumor site. The mean total administered activity per patient was 31.67 ± 6.57 MBq (range, 21.1–42.5 MBq).

**TABLE 2.** Residence Time of ^221^Fr and ^213^Bi in Source Organs and Lesions in Cycles 1 and 4 of Therapy

	Residence Time (h)				
RPT	Kidneys	Liver	Red bone marrow	Spleen	Lesion*
^221^Fr					
Cycle 1	2.10 ± 1.05 (0.79–4.32)	11.70 ± 6.65 (4.03–26.73)	0.67 ± 0.55 (0.10–1.91)	2.55 ± 1.33 (0.92–4.97)	2.64 ± 3.98 (0.06–15.32)
Cycle 4	2.43 ± 1.30) (1.05–5.48)	10.99 ± 5.35 (5.10–20.05)	0.57 ± 0.58 (0.08–1.90)	2.43 ± 0.86 (1.39–3.73)	1.62 ± 1.43 (0.17–6.16)
^213^Bi					
Cycle 1	3.76 ± 1.00 (2.27–5.61)	13.50 ± 5.82 (8.67–22.99)	1.11 ± 0.59 (0.49–2.33)	2.14 ± 1.12 (1.07–4.49)	2.02 ± 3.47 (0.06–15.91)
Cycle 4	3.86 ± 2.12 (2.22–9.31)	12.32 ± 5.50 (5.81–20.38)	0.96 ± 0.61 (0.33–2.15)	2.21 ± 0.84 (1.34–3.37)	1.57 ± 1.26 (0.19–5.45)

* Any single lesion.

Data represent mean ± SD, with range in parentheses.

### Quantitative Imaging of ^221^Fr and ^213^Bi

**FIGURE 4.**
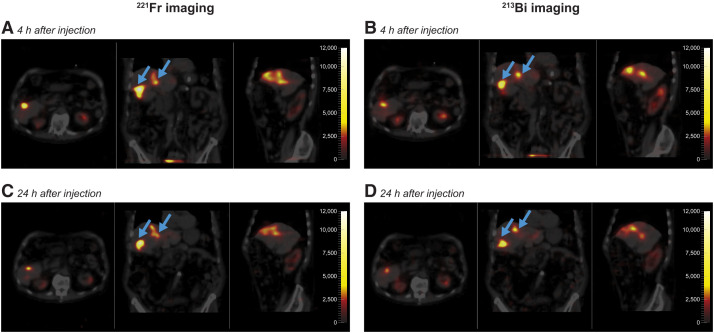
Example of quantitative reconstruction of ^221^Fr (A and C) and ^213^Bi (B and D) fused with CT images for representative patient (patient 3) at 4 (top) and 24 h postinjection (bottom). Arrows indicate tumor locations. SPECT images were acquired in 2 energy windows: 218.2 keV (±20%) to localize ^221^Fr and 440 keV (±20%) to localize ^213^Bi.

Figure 4 shows an example of quantitative reconstructions of ^221^Fr and ^213^Bi fused with CT images at 4 and 24 h after weight-based injection of [^225^Ac]Ac-DOTATATE 10.2 MBq in cycle 1 for a single representative patient (patient 3).

## DISCUSSION

The results of this dosimetry substudy of the global ACTION-1 trial demonstrated the feasibility of imaging and obtaining quantitative information appropriate for dosimetry of ^225^Ac, on the basis of SPECT/CT imaging of ^221^Fr (4.8-min half-life), the first daughter of and surrogate for ^225^Ac, and ^213^Bi (46.6-min half-life). ^213^Bi was directly and simultaneously quantified alongside ^221^Fr, with results suggesting that ^213^Bi mostly remained with the DOTATATE delivery agent in the tumor sites, with only a minor fraction of free ^213^Bi going to the kidneys (Table 2). This mitigates previous concerns that free ^213^Bi concentrates in the kidneys, significantly contributing to the radiation dose to the kidneys (28,29). These results indicate that, for [^225^Ac]Ac-DOTATATE, ^225^Ac daughters mostly remain with the delivery agent and decay at the same location as the parent. This observation, combined with the tumor and normal tissue absorbed doses seen in Figure 2A that indicate favorable tumor–to–normal tissue dose ratios, is supportive of [^225^Ac]Ac-DOTATATE for the treatment of patients with SSTR-expressing GEP-NETs.

The most frequently reported toxicities associated with radiolabeled SSAs are hematologic and renal (30). Administration of the recommended activity and number of cycles for [^177^Lu]Lu-DOTATATE (7.4 GBq every 8 wk for 4 doses) results in expected absorbed doses to the kidneys and red bone marrow of 19.4 Gy and 1.0 Gy, respectively (31). Assuming the fixed activity of 10.2 MBq of [^225^Ac]Ac-DOTATATE every 8 wk for 4 or fewer treatment cycles under investigation in phase 3 of ACTION-1 and the ADC_RBE=5_ reported in this substudy, the estimated absorbed doses to the kidneys and red bone marrow are 22.3 and 1.1 Gy, respectively, consistent with the absorbed doses expected for typical [^177^Lu]Lu-DOTATATE treatment.

Most tumor ADC_RBE=5_ values in cycle 4 were less than the corresponding tumor ADC_RBE=5_ in cycle 1. In addition, many tumors defined in cycle 1 were not identifiable in cycle 4. This supports the clinical efficacy observed in ACTION-1 phase 1b, in which 1 complete response per RECIST version 1.1 and 4 partial responses were reported for a confirmed overall response rate of 29.4% (32). The kidneys did not follow this trend, as the renal ADC_RBE=5_ was slightly increased in cycle 4, possibly because of the reduced tumor burden between cycles 1 and 4.

The linear energy transfer of α particles is approximately 400 times that of photons and β particles, leading to fundamentally different biologic effects. Accordingly, absorbed doses for different emission types must be weighted appropriately to reflect likely biologic effects. The RBE is the appropriate weighting factor for deterministic effects (e.g., tissue toxicity, tumor-kill efficacy), and a value of 5 was recommended in MIRD 22 (9). There is, however, substantial uncertainty regarding the most appropriate RBE for α-particle emission in radiopharmaceuticals. Evidence suggests that a single RBE value will not be applicable across all tumor or tissue types, and that the RBE will depend on the DNA double-strand break repair capacity of the irradiated cell (10). For this study, calculations were made using RBE of 5, which is aligned with the guidance in *MIRD Pamphlet No. 22* (9).

Some assumptions and limitations of this analysis should be considered. We assumed that activity distributions for ^225^Ac and ^217^At were the same as that of ^221^Fr and that the distribution of ^213^Bi daughters was the same as that of ^213^Bi. Given the short half-lives of ^217^At and ^213^Po, it is reasonable to assume that these radionuclides likely have the same activity distribution as their parents. The half-life of ^221^Fr is considerably smaller than the temporal resolution of the SPECT scan. The extent of ^221^Fr redistribution after ^225^Ac decay depends on the location of ^225^Ac when it decays. If bound in a cell, ^221^Fr would likely have the same activity distribution as ^225^Ac. Similarly, ^213^Bi and its α-emitting daughter ^213^Po likely have the same activity distribution. Other daughters make negligible contributions to overall absorbed doses.

Regarding limitations, the radiopharmaceutical solution likely contained some ^225^Ac daughters because of delays between production/purification and administration. Although daughter activities are almost identical to the activity of ^225^Ac after reaching equilibrium, the number of daughter atoms is expected to be small compared with the number of ^225^Ac atoms (<0.32%) because of half-life differences. The absorbed dose resulting from free daughter ions at injection was expected to be considerably smaller than that from the ^225^Ac decay chain (<0.14%, assuming total absorption). Therefore, as dose contribution from daughters in the injectate is negligible, their effects on dosimetry were not explicitly accounted for.

As this was the first study of its kind for ^225^Ac, a cautionary approach was taken regarding scanning: 60-min scans were recommended but scan duration was at the investigator’s discretion. Because of specialized reconstruction methods and long acquisition times, α-emitter imaging is not yet ready for routine patient implementation.

## CONCLUSION

Initial dosimetry data from the ACTION-1 study of [^225^Ac]Ac-DOTATATE suggest that ^221^Fr and ^213^Bi can be imaged separately and simultaneously by SPECT/CT. Results indicate a favorable tumor–to–normal tissue absorbed dose ratio, supportive of [^225^Ac]Ac-DOTATATE for the treatment of SSTR-expressing GEP-NETs. This work demonstrates the feasibility of performing image-based dosimetry and evaluating daughter isotope location for ^225^Ac-labeled radiopharmaceuticals at clinical levels where γ-emission is low. This method can assist in dosimetry of other ^225^Ac-labeled radiopharmaceuticals.

## DISCLOSURE

This study was supported by RayzeBio, Inc., which also funded medical writing and editorial support provided by Miller Medical Communications Ltd. George Sgouros reports being a consultant for Bayer, POINT Biopharma, PRECIRIX, RayzeBio, and Actinium Pharmaceuticals; participating in a scientific advisory board for Orano Med, Convergent Therapeutics, Nucleus RadioPharma, and RadioMedix; receiving grants from the National Institutes of Health; and being a founder of and stakeholder in Radiopharmaceutical Imaging and Dosimetry, LLC (Rapid). Gary Ulaner reports trial funding, consulting fees, and trial drugs and materials from RayzeBio. Bin He reports being employed by Radiopharmaceutical Imaging and Dosimetry, LLC (Rapid), and was contracted to perform the dosimetry calculations for the study described in the paper (payments made to Rapid). Michael Ghaly reports being a cofounder and board member of Radiopharmaceutical Imaging and Dosimetry, LLC (Rapid). Eric Frey reports being a founder and owner of Radiopharmaceutical Imaging and Dosimetry, LLC (Rapid), which received payments from RayzeBio to perform the dosimetry described in this publication; receiving royalties or licenses from Rapid/Johns Hopkins University; and being a member of the Society of Nuclear Medicine and Molecular Imaging Dosimetry Task Force (unpaid position). The software used for the quantitative reconstructions in this paper is derived from software licensed by Rapid from Johns Hopkins University. Eric Frey is one of the inventors of that software and shares in the royalties that Rapid pays to Johns Hopkins University. Denis Ferreira reports being employed by RayzeBio. Eileen Sneeden reports being employed by RayzeBio, Inc. Michael Morris reports being a board member and cofounder of United Theranostics and being a member of the board of advisors for Softhread. No other potential conflict of interest relevant to this article was reported.

## Notes

KEY POINTS **QUESTION:** Is [^225^Ac]Ac-DOTATATE dosimetry feasible? **PERTINENT FINDINGS:** These initial dosimetry data from the phase 1b/3 ACTION-1 clinical trial suggest that ^221^Fr and ^213^Bi can be used to image and obtain quantitative information appropriate for ^225^Ac using SPECT/CT and demonstrate that the daughter products of ^225^Ac remain with the delivery agent. A favorable tumor–to–normal tissue absorbed dose ratio was observed for [^225^Ac]Ac-DOTATATE. **IMPLICATIONS FOR PATIENT CARE:** These first dosimetry findings for ^225^Ac support the initial clinical findings from the ACTION-1 study, which demonstrate promising efficacy and safety/tolerability profiles for [^225^Ac]Ac-DOTATATE in patients with SSTR-expressing, well-differentiated GEP-NETs that progressed after ^177^Lu-labeled SSA therapy.
